# Selective cytotoxicity and antifungal properties of copper(II) and cobalt(II) complexes with imidazole-4-acetate anion or 1-allylimidazole

**DOI:** 10.1038/s41598-019-46224-6

**Published:** 2019-07-05

**Authors:** Katarzyna Gałczyńska, Karol Ciepluch, Łukasz Madej, Krystyna Kurdziel, Barbara Maciejewska, Zuzanna Drulis-Kawa, Aneta Węgierek-Ciuk, Anna Lankoff, Michał Arabski

**Affiliations:** 1 Department of Biochemistry and Genetics, Institute of Biology, Jan Kochanowski University, Świętokrzyska 15, 25-406 Kielce, Poland; 2 Holy Cross Oncology Center of Kielce, Artwińskiego 3, 25-734 Kielce, Poland; 3 Institute of Chemistry, Jan Kochanowski University, Świętokrzyska 15G, 25-406 Kielce, Poland; 4 Department of Pathogen Biology and Immunology, Institute of Genetics and Microbiology, University of Wroclaw, Przybyszewskiego 63-77, 51-148 Wrocław, Poland; 5 Department of Radiobiology and Immunology, Jan Kochanowski University, Świętokrzyska 15, 25-406 Kielce, Poland; 6 Center for Radiobiology and Biological Dosimetry, Institute of Nuclear Chemistry and Technology, Dorodna 16, 03-195 Warsaw, Poland

**Keywords:** Metals, Toxicology

## Abstract

The physicochemical properties of metal complexes determine their potential applications as antitumor agents. In this study, the antitumor properties of mononuclear cobalt(II) and copper(II) coordination compounds (stoichiometry: [Co(iaa)_2_(H_2_O)_2_]·H_2_O (iaa = imidazole-4-acetate anion), [Co(1-allim)_6_](NO_3_)_2_ (1-allim = 1-allylimidazole), [Cu(iaa)_2_H_2_O] and [Cu(1-allim)_4_(NO_3_)_2_]) and their ligands have been evaluated on human lung carcinoma A549 cells and normal bronchial BEAS-2B cells. Designing the chemical structure of new antitumor agents the possible interactions with macromolecules such as DNA or proteins should be take into account. PCR gene *tlr4* product served as DNA model, whereas lysozyme and phage-derived endolysin (both peptidoglycan degrading enzymes) were applied as protein/enzyme model. The interactions were analysed using PCR-HRM and circular dichroism, FT-IR, spectrophotometry, respectively. Additionally, the antimicrobial properties of the complexes at a non-cytotoxic concentration were analyzed against *S. aureus, E. coli, P. aeruginosa* and *C. albicans* strains. The results obtained in this study showed the selective cytotoxicity of metal complexes, mainly [Cu(1-allim)_4_(NO_3_)_2_] towards tumor cells. From all tested compounds, only [Co(iaa)_2_(H_2_O)_2_]^.^H_2_O non-covalently interacts with DNA. Cu(II) and Co(II) complexes did not affect the secondary conformation of tested proteins but modified the hydrolytic activity of enzymes (lysozyme and endolysin). Moreover, only [Co(iaa)_2_(H_2_O)_2_]^.^H_2_O exhibited the antifungal properties. In conclusion, Co(II) and Cu(II) metal complexes bearing two imidazole-4-acetate ligands seemed to be promising antitumor and antifungal agents for future drug design and application.

## Introduction

Transition metal complexes exhibit a wide range of antitumor and antimicrobial activities what combined with physicochemical properties determine their potential applications in clinical medicine^1–3^. Primarily, the discovery of cisplatin properties has revolutionized antitumor therapy^4,5^, whereas platinum (Pt)-based chemotherapy is related to Pt ability of DNA and proteins binding. Cisplatin and novel analogue complexes are still one of the most effective anticancer agents, however their usefulness is limited due to the relatively fast development of resistance in tumor cells and general adverse side effects in human body^6,7^. The design of chemical structure of new antitumor metal complexes should consider specific issues such as DNA binding mode and the interactions with proteins. One of the example of undesirable interactions regards to the serum albumin as the most abundant plasma protein distributed in the blood, which complex many metal ions in form of covalent bonds^6^. It strongly affects the pharmacokinetic properties and diminished the therapeutic dose transferred to tumor target position. Moreover, the presence of ligands in metals complexes might modulate the diffusion efficacy across the tumor cell membrane. Chelation reduces the polarity of metals and increases the lipophilic nature of the central metal atom, which in turns favours its permeation through the lipid layer of membrane^8^.

Taking into account above issues, the attention is currently focused mainly on the complexes coordination of copper Cu(II), cobalt Co(II), zinc Zn(II), ruthenium Ru(II) and nickel Ni(II) ions to form chemically stable chelates with predictable geometry. One of the example it the addition of aromatic N-containing ligands as imidazole derivatives^9,10^. The Cu(II) complexes with imidazole exhibit a different mechanism of action compared to cisplatin, binding to AT base pairs of DNA^11,12^. Moreover, it is assumed that Cu(II)-based complexes are less toxic for normal cells than to cancer cells. Cobalt(II) complexes have a more limited application compared to copper ones. Nevertheless, the Co(II) chelates with imidazole derivatives are also considered as a fertile ground for finding novel antitumor drugs^13,14^.

The Cu(II) and Co(II) metals might be also considered as antimicrobial agents. Their antibacterial properties depend on the penetration mechanisms through the bacterial cell membranes (diffusion *via* calcium or iron channels). Moreover, Co(II) might act as an antagonist of Fe(III) in the interaction with pyoverdine, a siderophore produced by *P. aeruginosa* reducing the ferric ions availability and uptake by bacterial cells^15^. Antibacterial action of Cu(II) ions is explained by two general mechanisms: (i) the change in bacterial membrane permeability due to the interaction between metal ions and outer membrane, and (ii) the interaction between bacteria and generated free radicals^16,17^. As previously reported, metals ions in complexes with imidazole derivatives are more effective in membrane destabilization than free ions, disturbing cell structural integrity and leading to microorganism eradication^18^. Antifungal properties of metals ions and their coordination complexes are connected with several defects in enzymatic steps of ergosterol biosynthesis. This is a major mechanism of metal complexes toxicity against fungal cells^19^.

Considering the need for new safe and effective antitumor agents, one of the main goals of this study was to evaluate the cytotoxic activity of four Co(II) and Cu(II) complexes having different number of imidazole derivatives (4-imidazoleacetic acid anion or 1-allylimidazole) on normal and carcinoma human cell lines. The antitumor effects were pre-evaluated by the measurements of metal complexes interactions with DNA and proteins. Additionally, the Co(II) and Cu(II) complexes were screened for their *in vitro* antibacterial and antifungal activities, at non-cytotoxic concentrations for eukaryotic cells.

## Results

In the first step of this study, the antitumor properties of [Cu(1-allim)_4_(NO_3_)_2_], [Co(1-allim)_6_](NO_3_)_2_, [Co(iaa)_2_(H_2_O)_2_]·H_2_O_,_ [Cu(iaa)_2_(H_2_O)] complexes and precursors (ligands and metal salt) were tested on carcinoma A549 cell line in comparison to normal BEAS-2B cells.

To identify the cellular target of metal complexes conditioning an antitumor activity, the interactions with DNA (using PCR-HRM technique) and proteins (lysozyme and recombinant endolysin) were tested using biophysical techniques. As the model DNA molecule the PCR gene *tlr4* product was used, whereas lysozyme and phage-derived endolysin served as protein/enzyme model. Lysozyme and phage endolysin degrade peptidoglycan (PG) in two different ways corresponding to their enzymatic specificity: muramidase or endopeptidase, respectively. Additionally, the antimicrobial properties of all tested metal complexes were analyzed only at non-cytotoxic concentrations to eukaryotic cells.

### Cytotoxic effect of metal complexes on eukaryotic cells lines

The cytotoxicity of tested complexes was determined using Annexin V-FITC apoptosis detection kit and MTT test. Tables 1 and 2 present the percentage of early/late apoptotic and necrotic A549 or BEAS-2B cells following the incubation with cobalt, cooper complexes or metal ions alone. The A549 cancer cells turned out to be more sensitive to Co(II) and Cu(II) ions alone than normal BEAS-2B ones. Interestingly, this tendency was statistically more significant in the case of coordination complexes of metals, especially [Co(iaa)_2_(H_2_O)_2_]^.^H_2_O and [Cu(iaa)_2_(H_2_O)]. Those complexes were able to induced the late apoptosis/necrosis at 250 μM in A549 cells in contrast to non-sensitive normal BEAS-2B cells (Table 1). [Cu(1-allim)_4_(NO_3_)_2_] generally possess stronger cytotoxic properties than copper ions alone and at the concentration above 60 μM and 125 μM exhibited the toxic activity on A549 cells and BEAS-2B, respectively. The ligands alone had no cytotoxic activity against both tested eukaryotic cell lines.

**Table 1:** Percentage of early and late apoptotic and necrotic A549 or BEAS-2B cells following treatment with cobalt complexes measured by flow cytometry; mean of three independent experiments ± SD.

Substance [μM]	A549 cells				BEAS-2B cells			
	Normal cells (Annexin−/IP−)	Apoptosis		Necrosis (Annexin−/IP+)	Normal cells (Annexin−/IP−)	Apoptosis		Necrosis (Annexin/IP+)
		Early (Annexin+/IP−)	Late (Annexin+/IP+)			Early (Annexin+/IP)	Late (Annexin+/IP+)	
Control	96.80 ± 0.92	0.52 ± 1.01	2.17 ± 0.33	1.10 ± 0.61	96.08 ± 2.02	1.53 ± 1.01	1.38 ± 0.17	0.98 ± 0.33
15 [Co(iaa)_2_(H_2_O)_2_]^.^H_2_O	96.60 ± 0.42	0.45 ± 0.07	1.65 ± 0.07	0.95 ± 0.35	96.60 ± 0.42	1.40 ± 0.01	1.15 ± 0.49	0.80 ± 0.01
15 [Co(1-allim)_6_](NO_3_)_2_	93.90 ± 1.99	0.90 ± 0.28	3.15 ± 0.92	2.05 ± 0.35	94.35 ± 2.62	2.90 ± 1.70	1.95 ± 1.20	0.70 ± 0.28
15 CoCl_2_	94.10 ± 2.12	0.35 ± 0.21	2.85 ± 0.92	2.75 ± 1.34	95.40 ± 2.26	2.05 ± 1.63	1.30 ± 0.57	1.30 ± 0.14
30 [Co(iaa)_2_(H_2_O)_2_]^.^H_2_O	96.70 ± 0.71	0.40 ± 0.00	1.80 ± 0.28	1.15 ± 0.35	96.65 ± 0.49	1.20 ± 0.28	1.10 ± 0.28	1.00 ± 0.28
30 [Co(1-allim)_6_](NO_3_)_2_	96.00 ± 0.70	0.75 ± 0.07	1.50 ± 0.42	1.75 ± 0.35	94.20 ± 2.97	3.55 ± 2.76	1.55 ± 0.78	0.70 ± 0.57
30 CoCl_2_	95.10 ± 1.98	0.30 ± 0.14	2.90 ± 0.99	1.70 ± 1.13	94.15 ± 1.48	2.80 ± 1.70	1.95 ± 0.07	1.10 ± 0.34
60 [Co(iaa)_2_(H_2_O)_2_]^.^H_2_O	96.65 ± 0.49	0.40 ± 0.01	1.35 ± 0.21	1.60 ± 0.70	96.90 ± 0.70	0.95 ± 0.35	1.10 ± 0.42	1.05 ± 0.07
60 [Co(1-allim)_6_](NO_3_)_2_	96.65 ± 0.49	0.65 ± 0.07	0.85 ± 0.07	1.85 ± 0.49	96.00 ± 1.13	1.90 ± 0.99	1.10 ± 0.01	1.05 ± 0.07
60 CoCl_2_	96.10 ± 1.27	0.15 ± 0.07	2.40 ± 0.42	2.35 ± 0.92	94.05 ± 2.33	3.30 ± 2.26	1.55 ± 0.21	1.10 ± 0.28
125 [Co(iaa)_2_(H_2_O)_2_]^.^H_2_O	**86.95** ± **0.64**	0.55 ± 0.07	**7.95** ± **2.62**	**4.55** ± **1.91**	96.15 ± 0.21	1.20 ± 0.14	1.15 ± 0.07	1.40 ± 0.14
125 [Co(1-allim)_6_](NO_3_)_2_	**50.40** ± **4.67**	0.55 ± 0.35	**27.15** ± **6.43**	**21.90** ± **1.41**	96.15 ± 1.34	1.95 ± 1.06	1.05 ± 0.35	0.85 ± 0.07
125 CoCl_2_	**17.25** ± **12.80**	0.25 ± 0.07	**60.70** ± **5.23**	**21.85** ± **7.57**	93.60 ± 0.42	2.90 ± 0.28	1.65 ± 0.64	1.80 ± 0.85
250 [Co(iaa)_2_(H_2_O)_2_]^.^H_2_O	**5.40** ± **4.10**	0.80 ± 0.14	**55.90** ± **0.42**	**37.90** ± **4.81**	92.70 ± 2.12	2.20 ± 0.14	2.95 ± 1.91	2.15 ± 0.17
250 [Co(1-allim)_6_](NO_3_)_2_	**3.15** ± **1.63**	2.65 ± 1.48	**80.35** ± **5.44**	13.80 ± 8.63	**91.80** ± **0.99**	2.60 ± 0.70	3.20 ± 1.56	**2.40** ± **0.14**
250 CoCl_2_	**4.10** ± **4.38**	0.20 ± 0.01	**72.00** ± **27.57**	**23.65** ± **3.26**	93.35 ± 1.34	1.90 ± 2.40	1.60 ± 0.01	3.15 ± 1.06
500 [Co(iaa)_2_(H_2_O)_2_]^.^H_2_O	**2.45** ± **0.78**	0.25 ± 0.01	**52.90** ± **12.30**	**44.40** ± **11.46**	91.10 ± 2.97	1.65 ± 0.07	3.60 ± 2.69	3.70 ± 2.14
500 [Co(1-allim)_6_](NO_3_)_2_	**4.95** ± **0.07**	**6.45** ± **1.63**	**55.85** ± **15.77**	**32.75** ± **17.47**	**2.50** ± **1.84**	0.60 ± 0.28	**79.40** ± **20.79**	17.45 ± 19.16
500 CoCl_2_	**1.90** ± **1.41**	0.40 ± 0.42	**75.10** ± **17.25**	22.60 ± 19.09	**59.65** ± **10.54**	3.10 ± 3.68	**23.50** ± **23.05**	13.75 ± 16.19

IP – propidium iodide. Bolded font when p < 0.05 in comparison to proper control (normal cells).

**Table 2:** Percentage of early and late apoptotic and necrotic A549 or BEAS-2B cells following treatment with copper complexes measured by flow cytometry; mean of three independent experiments ± SD. IP – propidium iodide.

Substance [μM]	A549				BEAS-2B			
	Normal cells (Annexin−/IP−)	Apoptosis		Necrosis (Annexin−/IP+)	Normal cells (Annexin−/IP−)	Apoptosis		Necrosis (Annexin−/IP+)
		Early (Annexin+/IP−)	Late (Annexin+/IP+)			Early (Annexin+/IP−)	Late (Annexin+/IP+)	
Control	97.60 ± 0.71	1.45 ± 0.42	0.68 ± 0.25	0.30 ± 0.08	94.50 ± 1.12	3.08 ± 0.87	1.65 ± 0.95	0.78 ± 0.46
15 [Cu(iaa)_2_(H_2_O)]	97.55 ± 0.21	1.15 ± 0.35	0.80 ± 0.14	0.55 ± 0.35	92.10 ± 0.42	4.70 ± 0.99	2.45 ± 1.77	0.80 ± 0.28
15 [Cu(1-allim)_4_](NO_3_)_2_	97.00 ± 1.70	1.30 ± 0.42	1.45 ± 0.92	0.30 ± 0.28	94.65 ± 0.46	3.05 ± 0.46	1.25 ± 0.47	0.80 ± 0.53
15 CuCl_2_	98.10 ± 0.14	1.00 ± 0.01	0.65 ± 0.21	0.25 ± 0.07	92.35 ± 2.19	4.55 ± 0.92	2.30 ± 1.98	0.80 ± 0.71
30 [Cu(iaa)_2_(H_2_O)]	97.10 ± 0.14	0.95 ± 0.35	1.50 ± 0.14	0.40 ± 0.28	92.80 ± 1.27	3.60 ± 0.14	2.80 ± 1.98	0.80 ± 0.57
30 [Cu(1-allim)_4_](NO_3_)_2_	95.10 ± 1.98	0.45 ± 0.07	6.55 ± 1.63	1.65 ± 0.35	94.43 ± 1.21	2.73 ± 0.85	2.03 ± 0.75	1.20 ± 0.98
30 CuCl_2_	97.75 ± 0.64	1.00 ± 0.01	0.95 ± 0.21	0.25 ± 0.07	93.20 ± 3.68	4.00 ± 1.13	2.40 ± 2.55	0.50 ± 0.14
60 [Cu(iaa)_2_(H_2_O)]	94.75 ± 0.49	0.45 ± 0.07	3.30 ± 0.42	1.45 ± 1.06	93.70 ± 1.13	2.60 ± 0.28	2.65 ± 2.05	1.05 ± 0.64
60 [Cu(1-allim)_4_](NO_3_)_2_	**68.05** ± **5.16**	0.20 ± 0.01	**26.35** ± **8.13**	**5.40** ± **2.97**	94.40 ± 1.49	2.13 ± 0.55	2.13 ± 0.93	1.37 ± 0.83
60 CuCl_2_	94.75 ± 0.07	0.45 ± 0.07	3.25 ± 0.07	1.60 ± 0.14	93.55 ± 2.62	3.35 ± 0.78	2.45 ± 1.91	0.65 ± 0.07
125 [Cu(iaa)_2_(H_2_O)]	**88.45** ± **2.33**	0.10 ± 0.01	**7.20** ± **0.42**	**4.20** ± **2.83**	94.20 ± 0.70	2.00 ± 0.42	2.50 ± 0.14	1.25 ± 0.35
125 [Cu(1-allim)_4_](NO_3_)_2_	**1.15** ± **1.48**	0.05 ± 0.07	**53.30** ± **30.91**	**45.50** ± **28.47**	**67.77** ± **5.58**	3.93 ± 1.94	**24.20** ± **2.98**	4.10 ± 3.73
125 CuCl_2_	**79.60** ± **1.13**	0.15 ± 0.07	**9.50** ± **2.69**	**10.75** ± **3.75**	94.55 ± 1.77	2.65 ± 0.78	2.35 ± 1.20	0.55 ± 0.21
250 [Cu(iaa)_2_(H_2_O)]	**17.25** ± **7.71**	0.05 ± 0.07	**14.72** ± **1.63**	**67.95** ± **6.15**	94.50 ± 0.14	1.35 ± 0.21	3.05 ± 0.78	1.10 ± 0.42
250 [Cu(1-allim)_4_](NO_3_)_2_	**0.00** ± **0.00**	0.00 ± 0.00	**23.45** ± **7.42**	**77.50** ± **6.08**	**7.33** ± **8.75**	1.87 ± 1.77	**77.97** ± **18.51**	**12.87** ± **6.00**
250 CuCl_2_	**9.95** ± **4.88**	0.00 ± 0.00	**23.50** ± **14.13**	**66.50** ± **10.32**	94.15 ± 1.06	2.10 ± 0.71	2.75 ± 0.92	0.95 ± 0.64
500 [Cu(iaa)_2_(H_2_O)]	**0.60** ± **0.28**	0.00 ± 0.00	**15.15** ± **5.87**	**84.25** ± **6.15**	94.85 ± 1.63	0.70 ± 0.28	1.75 ± 0.78	2.75 ± 0.49
500 [Cu(1-allim)_4_](NO_3_)_2_	**9.55** ± **1.34**	1.90 ± 0.57	**17.15** ± **8.96**	**71.40** ± **10.18**	**1.43** ± **0.76**	1.90 ± 0.30	**79.77** ± **14.88**	**16.93** ± **9.20**
500 CuCl_2_	**0.15** ± **0.07**	0.00 ± 0.00	**10.75** ± **0.64**	**89.05** ± **0.49**	**84.80** ± **1.41**	1.30 ± 0.99	**12.35** ± **1.48**	1.55 ± 1.06

Bolded font when p < 0.05 in comparison to proper control (normal cells).

**Figure 1:**
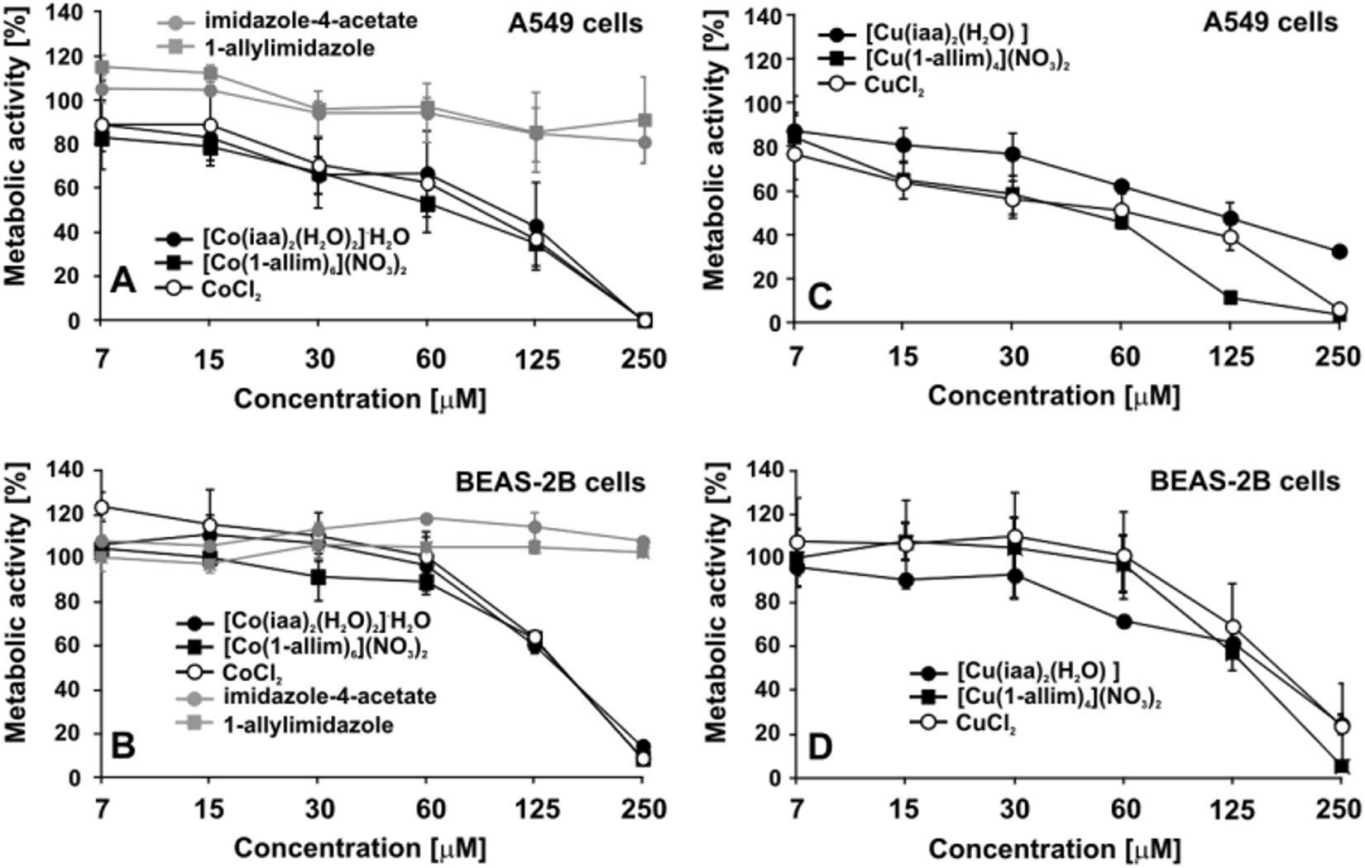
The MTT percentage of A549 and BEAS-2B cells metabolic activity under an increasing dose of [Co(iaa)_2_(H_2_O)_2_]H_2_O, [Co(1-allim)_6_](NO_3_)_2_, [Cu(iaa)_2_(H_2_O)], [Cu(1-allim)_4_(NO_3_)_2_] complexes or their metals and ligands alone after 48 hours treatment.

Figure 1 shows the metabolic activity of A549 and BEAS-2B cells after the incubation with [Co(iaa)_2_(H_2_O)_2_]·H_2_O, [Co(1-allim)_6_](NO_3_)_2_, [Cu(iaa)_2_(H_2_O)], [Cu(1-allim)_4_(NO_3_)_2_] complexes or their metals or ligands alone at the range of 7–250 μM concentrations measured by the MTT assay. The results showed that: (i) A549 cells are more sensitive to Co(II) and Cu(II) complexes or their ions alone compared to the normal BEAS-2B cells; (ii) [Cu(1-allim)_4_(NO_3_)_2_] is more cytotoxic than copper ions alone; and (iii) ligands are non-cytotoxic for both tested eukaryotic cell lines.

### DNA binding by metal complexes

**Figure 2:**
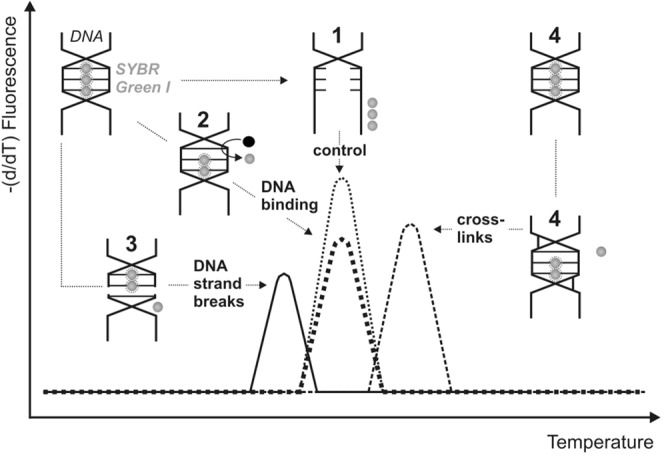
The interpretation of metals interactions with DNA measured by PCR-HRM. (1) the double stranded DNA melts apart and releasing fluorescence dye away; (2) metal (•) interacts with DNA and displaces SYBR Green I; (3) metal induces DNA strand breaks and shortened amplicon releasing lower amount of fluorescence dye away at lower temperature in comparison to control (1); (4) metal binds with DNA formed cross-links and displaces fluorescence dye at higher temperature than control probe.

The interaction of metal complexes with DNA molecule as a common target of antitumor agents, was analysed using PCR-HRM and cisplatin was used as the positive control. In this experiment we used a high resolution melting (HRM) procedure (PCR-based technique), to analyse the melting temperature of amplicon/tested chemical agent complex formed after DNA incubation with metal complexes. This application of PCR-HRM technique was described in detail in our previously work^20^. The analysed agents might interact with DNA intercalated by SYBR Green I dye. The chemical compounds affecting the DNA structure result in the changes of DNA melting temperature in comparison to the control (untreated DNA). That event might be observed by showing the course of fluorescence vs. temperature, generating a melting curve specific to the type of DNA-metal interaction (Fig. 2). In our study cisplatin has been chosen as a positive control having a well-known DNA binding mechanism of creating the intra-strand and inter-strand DNA cross-links^21^.

**Figure 3:**
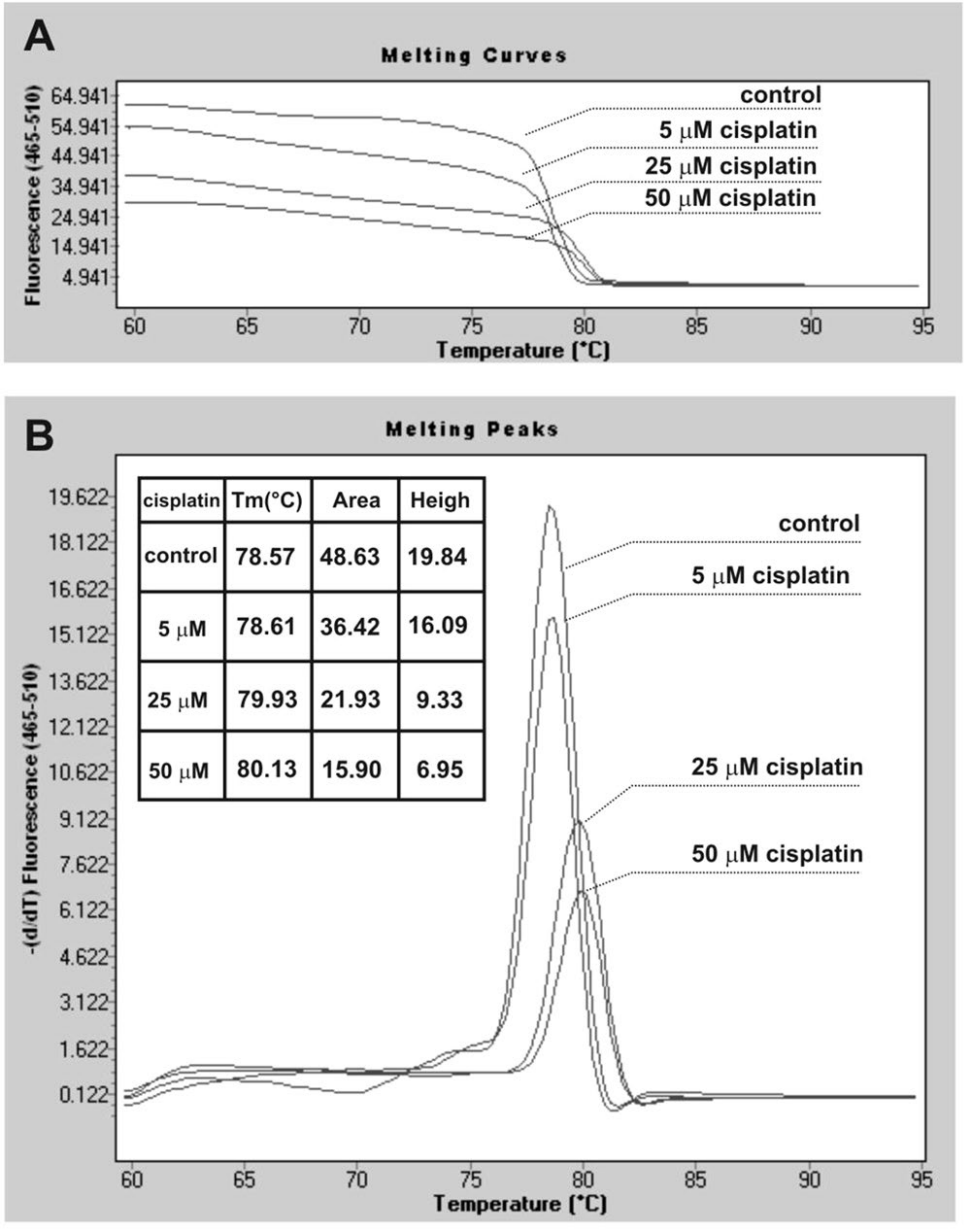
The melting curve of DNA after incubation with cisplatin for 1 h at 37 °C analyzed by LightCycler 480 Gene Scanning Software (version 1.5).

Figure 3 shows the melting curve of DNA after the incubation with cisplatin for 1 h at 37 °C. DNA cross-linked with cisplatin (displace of the SYBR Green I molecules corresponds to the lower level of fluorescence) requires a higher temperature for its denaturation in comparison to the free DNA control. The melting peak was shifted to a higher temperature range in a dose-depended manner. The lower peak is observed on the melting curve of DNA incubated with [Co(iaa)_2_(H_2_O)_2_]·H_2_O (Table 3). It means that only [Co(iaa)_2_(H_2_O)_2_]·H_2_O binds non-covalently to DNA and displaces the SYBR Green I from the minor groove of DNA by electrostatic interactions. The antitumor activity of this complex could be additionally associated with a DNA-binding mode.

**Table 3:** The melting curve (peak) properties of DNA after incubation with Cu(II) or Co(II) metal complexes for 1 h at 37 °C analyzed by LightCycler 480 Gene Scanning Software (version 1.5).

Substance	Tm [°C]	Area	Heigh
control	80.69	11.47	4.27
[Cu(1-allim)_4_(NO_3_)_2_]	81.10	10.92	3.60
[Cu(iaa)_2_(H_2_O)]	81.09	10.96	3.90
[Co(1-allim)_6_](NO_3_)_2_	80.93	9.57	3.76
[Co(iaa)_2_(H_2_O)_2_]H_2_O	80.80	4.85	1.74

### Interaction of proteins with Cu(II) and Co(II) metal complexes

**Figure 4:**
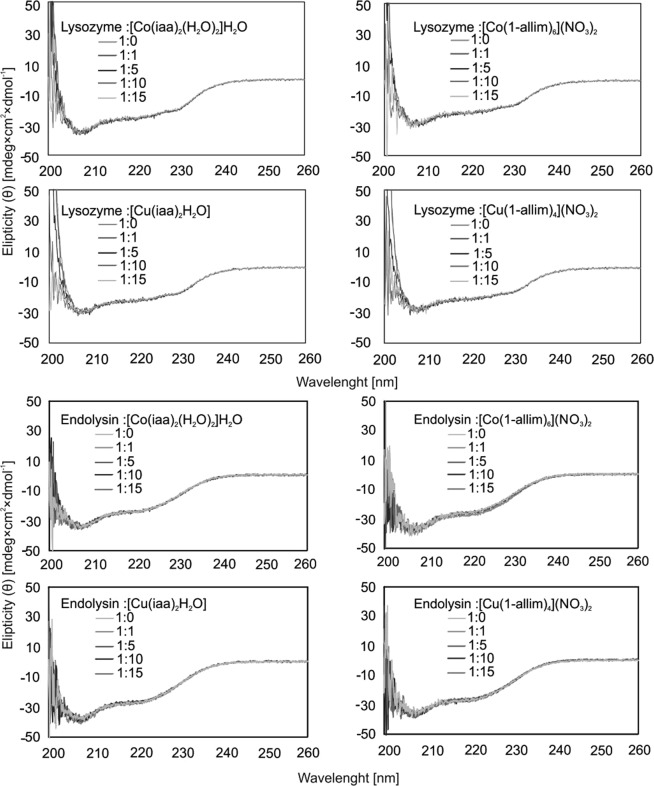
CD spectra of the lysozyme (**A**) and endolysin (**B**) in the presence of [Co(iaa)_2_(H_2_O)_2_]H_2_O, [Co(1-allim)_6_](NO_3_)_2_, [Cu(iaa)_2_(H_2_O)] and [Cu(1-allim)_4_(NO_3_)_2_] at molar ratio in range 1:1 to 1:50 (protein:complex respectively) measured in the far-UV region (the range between 260 and 195 nm). The experiments were done in 10 mM sodium phosphate buffer (pH 7.4).

Phage recombinant endolysin (KP27 endopeptidase) and commercially available lysozyme were chosen as an enzyme model for interactions with Cu(II) and Co(II) metal complexes analysis. The changes in the secondary structure of enzymes by metal complexes (measured by circular dichroism, CD) were determined in correlation to PG degradation (measured by spectrophotometry). The secondary structure of endolysin and lysozyme was measured in the wavelength range from 260 to 195 nm (Fig. 4). In the CD spectra, no changes in the secondary structure were observed for endolysin and lysozyme, at any molar ratio of metal complexes presence (Fig. 4).

**Figure 5:**
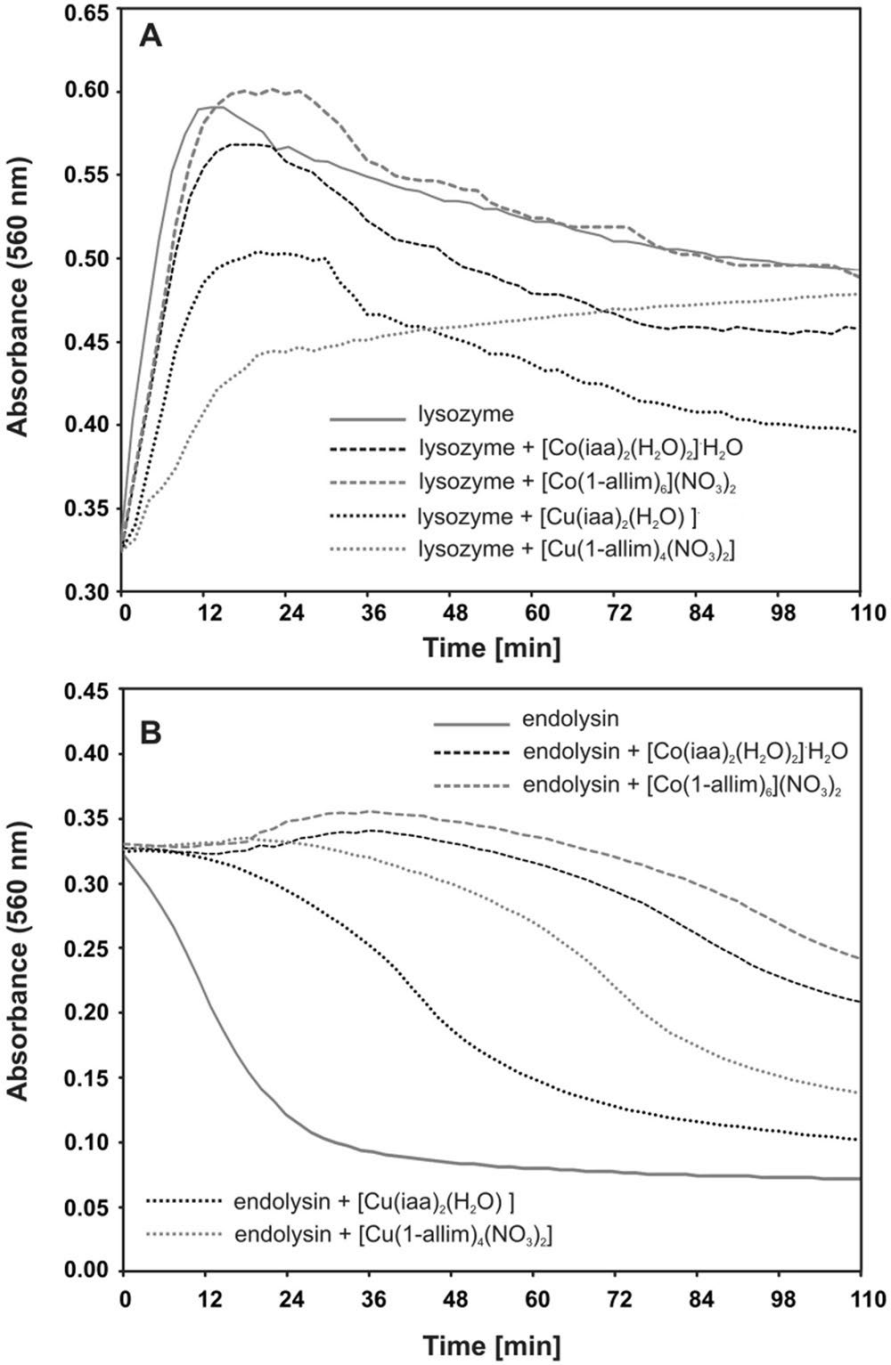
The spectrophotometric analysis of peptidoglycan degradation by lysozyme (**A**) or endolysin (**B**) in the presence of [Co(iaa)_2_(H_2_O)_2_]^.^H_2_O, [Co(1-allim)_6_](NO_3_)_2_, [Cu(iaa)_2_(H_2_O)] and [Cu(1-allim)_4_(NO_3_)_2_] at 1:5 molar ratio (protein:complex respectively) measured at 560 nm for 110 min with 2 min time intervals at 37 °C.

Additionally, we have tested PG degradation by selected enzymes after the incubation with metal complexes. The maximum of PG degradation occurred 15–20 minutes after lysozyme (lysozyme:complex) administration (Fig. 5). Because PG is not soluble in water, the maximum of absorbance showed up after 20 minutes and decreased very slowly. Not significant difference was observed in lysozyme and lysozyme:complexes activity, except for [Cu(1-allim)_4_(NO_3_)_2_], where the absorbance was still increasing (Fig. 5A). The results presented in Fig. 5B showed that the activity of endolysin decreased in the function of time in the presence of all metal complexes. The decay of the absorbance started immediately from 0.24 to 0.073 after 20 min (control). In the presence of metal complexes ([Cu(iaa)_2_H_2_O] and [Cu(1-allim)_4_(NO_3_)_2_] the decay of absorbance to the constant level started after 10 min and stopped around after 40–50 min. In the presence of [Co(1-allim)_6_](NO_3_)_2_, [Co(iaa)_2_(H_2_O)_2_]·H_2_O the absorbance started decaying after 30 min and stoped decaying after 60 min. The results suggest the stronger inhibition of endolysin activity in the presence of Cu(II) metal complexes compared to Co(II) complexes.

It means that generally all tested complexes changed the kinetics or inhibited the enzymatic activity of endolysin. In the case of endolysin, Cu(II) and Co(II) complexes with imidazole-4-acetate anion or 1-allylimidazole had a negative impact on enzymatic activity. Above results suggest a completely different mechanism of metal complex interactions with the molecule surface of these two proteins affecting catalytic site functioning^22^.

### Antimicrobial properties of metal complexes

**Figure 6:**
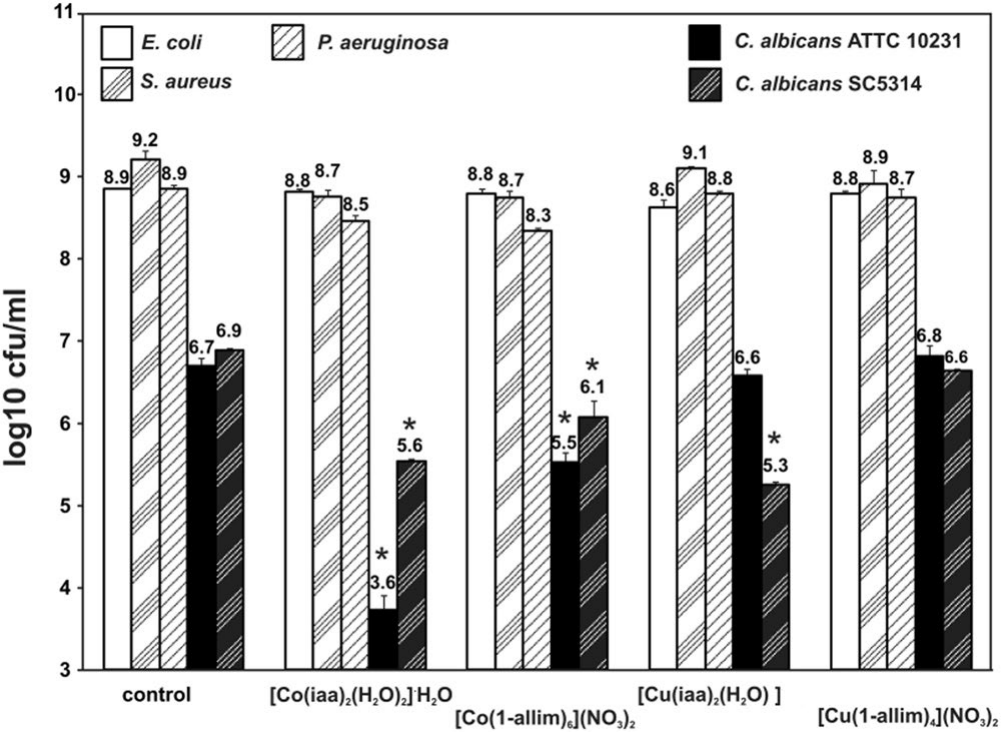
The antimicrobial properties of metal complexes at non-cytotoxic concentrations against eukaryotic cells: 60 µM for [Co(iaa)_2_(H_2_O)_2_]^.^H_2_O, [Co(1-allim)_6_](NO_3_)_2_ and 30 µM for [Cu(iaa)_2_(H_2_O)], [Cu(1-allim)_4_(NO_3_)_2_]_._ The log10 cfu/ml denoted.

Figure 6 shows the antimicrobial effect of metal complexes at non-cytotoxic concentrations against bacterial culture of *E. coli, S. aureus*, *P. aeruginosa* and *Candida albicans* cells. Generally, the [Co(iaa)_2_(H_2_O)_2_]^.^H_2_O, [Co(1-allim)_6_](NO_3_)_2_, [Cu(iaa)_2_(H_2_O)] and [Cu(1-allim)_4_(NO_3_)_2_] complexes had weak antibacterial activity in contrast to antifungal effect, where mainly [Co(iaa)_2_(H_2_O)_2_]^.^H_2_O reduced log10 cfu/ml from 6.7 to 3.6 (<99.9%) for *C. albicans* ATTC 10231 and from 6.9 to 5.6 (96%) for *C. albicans* SC5314. The [Co(iaa)_2_(H_2_O)_2_]^.^H_2_O had a stronger effect against fungal strains than components of this complex alone (results not shown).

## Discussion

The antitumor and antimicrobial properties of metal complexes are investigated for decades. Depending on the metal type in complex, the different biological properties can be observed. In this study, the Cu(II) and Co(II) complexes were tested. The antitumor activity was observed for Cu-complexes in contrast to none for Co-complexes. The results showed that [Cu(1-allim)_4_(NO_3_)_2_] at 125 µM concentration has selective cytotoxic properties towards tumor cells, higher than metals alone. No cytotoxic effect was detected for normal cell line treated at this concentration of metal complexes.

For cytotoxicity analyses we have used A549 tumor and BEAS-2B normal cell lines. The choice of model cell lines was made on the basis of previous report^23^. The authors characterized the expression of 380 genes encoding proteins involved in the metabolism of xenobiotics in commonly used lung cell lines and four primary cultures of human bronchial epithelial cells. This group of genes comprised 137 genes of phase I enzymes (including 56 P450s), 69 genes of phase II enzymes, 103 genes of transporters (including 31 ABC and 62 SLC transporters), 48 genes of nuclear receptors and transcription factors (including coactivators and corepressors), and 23 miscellaneous genes (including 9 metallothioneins). As the BEAS-2B cell line shows the highest homology in the gene expression pattern compared to the primary cells and the lowest number of dysregulated genes with nontumoral lung tissues, it could be used as a model for toxicological and pharmacological studies^24^. The A549 cells were chosen for this study as the most frequently used experimental model of tumor line in similar studies^25,26^.

There are several potential mechanisms of antitumor activity of metal complexes, especially in the case of Cu-complexes. These types of activity could be associated with the transport of Cu(II) ions into the cell. The Cu(II) ions are introduced by specific copper transporters hCtr in the Cu(I) form. The presence of natural copper transport system is crucial from the clinical point of view because no additional exogenous drugs carriers is need. Thus, the cellular effects of metal complexes activity might be determined by their interaction with proteins (eg. receptors) or nucleic acids. Considering metal complexes activity not only the interactions with DNA but also with proteins should be analyzed as the targeted molecules in antitumor mechanisms. In our study, lysozyme and endolysin, two low molecular proteins were chosen as an protein/enzyme model. All tested complexes have no influence on the secondary structure of lysozyme and endolysin, but they selectively affected the activity of studied proteins. Only Cu-complexes decreased the activity of both enzymes suggesting the interaction with the catalytic center of enzymes. That might explain the specific ability of [Cu(1-allim)_4_(NO_3_)_2_] in antitumor activity.

The cytotoxicity of metal complexes can also rely on the interaction with DNA like observed for cisplatin^21^. However, in our case the Cu-complexes are not able to bind to DNA. Only [Co(iaa)_2_(H_2_O)_2_]^.^H_2_O exhibited that tendency, but no antitumor activity was noticed.

The next tested property of metal complexes was the antimicrobial activity. There was no antibacterial effect seen after metal complexes treating. The antifungal properties were observed mainly for [Co(iaa)_2_(H_2_O)_2_]H_2_O complex at non-cytotoxic concentrations for eukaryotic cell lines. The probable antifungal mode of action might be related to a metal complex-protein interactions. Proteins that play role in the lipid metabolism, cell wall biogenesis, protein metabolism, electron transport, ATP synthesis and cellular stress protein samples were indentified in the lipid rafts of *C. albicans* located on the cell surface^27^. These proteins might be targeted and modified by metal complexes.

Kasuga *et al*. as well as Nawar *et al*. found that mainly the chemically labile complex, which has ability to the ligand replacement, exhibits a bactericidal effect. Such effect is determined by the substitution reactions of released ligands or metal from their complex with the main residues of targeted bacterial cell components^28,29^. It means that the stable chemical structure of metal complexes used in our study limited their bactericidal effect.

In conclusion, this pilot study showed that [Cu(1-allim)_4_(NO_3_)_2_] has promising antitumor properties but it needs further investigations. Additionally, the antifungal properties of [Co(iaa)_2_(H_2_O)_2_]^.^H_2_O present a potential application from clinical point of view.

## Material and Methods

### Metal complexes and their synthesis

**Figure 7:**
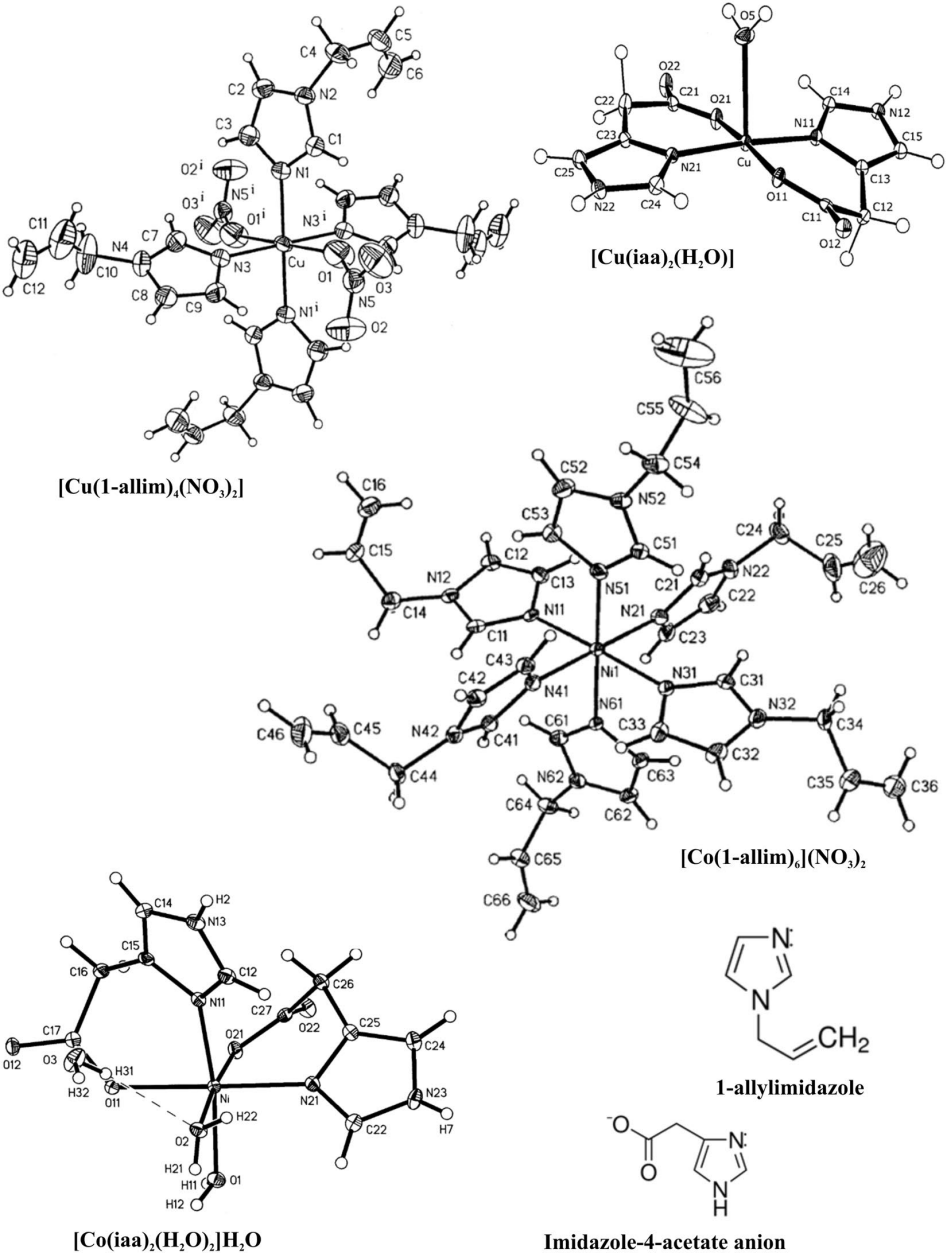
Chemical structures of tested compounds and their ligands alone.

The study includes the coordination compounds of Co(II) and Cu(II) with 1-allylimidazole (1-allim) or imidazole-4-acetate anion (iaa) (Fig. 7). The 1-allylimidazole complexes are described by the following formulas: [Cu(1-allim)_4_(NO_3_)_2_] and [Co(1-allim)_6_](NO_3_)_2_. The molecules of imidazole-4-acetate anion (iaa) compounds are described by the following formulas [Co(iaa)_2_(H_2_O)_2_]·H_2_O and [Cu(iaa)_2_(H_2_O)]. These complexes were previously characterized through their crystal structure and physical-chemical properties (IR, farIR, UV-Vis-NIR spectra, magnetic moment and molar conductivity) by our team^30–32^ and Drożdżewski *et al*.^33^.

The synthesis of [Cu(1-allim)_4_(NO_3_)_2_] and [Co(1-allim)_6_](NO_3_)_2_: to a solution of 1 mmol of Cu(NO_3_)_2_·3H_2_O or Co(NO_3_)_2_·6H_2_O dissolved in a mixture of propan-2-ol and trimethyl orthoformate (1:1 v/v), a solution of 6 mmol of 1-allylimidazole in the same solvent mixture was added. The crude products, which precipitate after few days, were recrystallized from propan-2-ol^30,32^.

The synthesis of [Co(iaa)_2_(H_2_O)_2_]·H_2_O and [Cu(iaa)_2_H_2_O]: the complexes of cobalt(II) and copper(II) with imidazole-4-acetate anion were obtained from aqueous solutions containing Co(NO_3_)_2_·6H_2_O or CuSO_4_·5H_2_O and sodium salt imidazole-4-acetic acid (Na(4-iaa)·H_2_O) of the molar ratio 1:2. Raspberry-red cobalt(II) complexes and dark blue crystals of copper(II) ones were dried in the air^31,33^.

The 1-allylimidazole, sodium salt imidazole-4-acetic acid (Na(4-iaa)·H_2_O), Co(NO_3_)_2_·3H_2_O, CuSO_4_·5H_2_O, propan-2-ol and trimethyl orthoformate were purchased from Aldrich Chemical Co. and were used without further purification, while the solvents used were RPE grade.

### Bacterial strains and eukaryotic cell lines

*Escherichia coli* ATCC8739*, Staphylococcus aureus* ATCC6538P*, Pseudomonas aeruginosa* ATCC15692 (PAO1), *Candida albicans* ATTC10231 and *Candida albicans* SC5314 strains originated from Jan Kochanowski University, Poland collection. The eukaryotic cell lines: adenocarcinoma human alveolar basal epithelial A549 cells (ATCC® CCL-185™) and normal human bronchial epithelium BEAS-2B cells (ATCC® CRL-9609™) were provided by American Tissue Cell Collection. A549 cells were cultured in F-12K medium (Sigma–Aldrich Chemicals, USA) supplemented with 10% fetal calf serum (Invitrogen, CA, USA), 2 mM L-glutamine (Sigma–Aldrich Chemicals, USA) and antibiotics (100 units/ml penicillin and 100 µg/ml streptomycin (Invitrogen, CA, USA) at 37 °C. BEAS-2B cells were cultured in LHC9 medium (Invitrogen, CA, USA) at 37 °C. Both cell lines were cultured in a humidified 5% CO_2_ atmosphere.

### Recombinant endolysin preparation

The recombinant phage-borne endolysin was prepared according to the method described previously by Maciejewska *et al*.^34^. Briefly, the coding sequence for *Klebsiella* phage KP27 endopeptidase was cloned into the commercially available pEXP-5-CT/TOPO® TA expression vector (Invitrogen, Thermo Fisher Scientific, Waltham, MA, USA) according to the manufacturer recommendations, and BL21 (DE3) pLysS (Agilent Technologies, Santa Clara, CA, USA) was transformed with the expression construct. The expression was induced by the addition of isopropyl-β-D-thiogalactopyranoside (IPTG, final concentration of 0.5 mM), and bacteria further culture additional 18 h at 20 °C. The recombinant protein was purified from the filtered supernatant by affinity chromatography using NGC Medium Pressure Chromatography Systems (Bio-Rad, Hercules, CA, USA) combined with 5-ml nickel columns: Bio-Scale Mini Profinity IMAC Cartridges (Bio-Rad, Hercules, CA, USA) and dialyzed against PBS buffer. The concentration of purified recombinant enzyme was then determined fluorimetrically (Qubit® Protein Assay Kit, Molecular Probes, Thermo Fischer Scientific, Waltham, MA, USA).

### Analysis of cytotoxicity of metal complexes on eukaryotic cells

The human lung normal BEAS-2B and carcinoma A549 were selected to compare the antitumor activity of four tested Co(II) and Cu(II) complexes with imidazole derivatives using Annexin V-FITC apoptosis detection test.

The BEAS-2B cells exhibited the highest homology in gene expression pattern with primary cells and the lowest number of dysregulated genes compared with non-tumoral lung tissues (comparison of the expression profiles of 380 genes encoding proteins involved in the metabolism of xenobiotics in 10 commonly used lung cell lines and four primary cultures of human bronchial epithelial cells)^23^. A549 was used as a model of the cancer cell line. The exponentially growing A549 and BEAS-2B cells were treated for 48 hours with [Co(iaa)_2_(H_2_O)_2_]H_2_O, [Co(1-allim)_6_](NO_3_)_2_, [Cu(iaa)_2_(H_2_O)], [Cu(1-allim)_4_(NO_3_)_2_] complexes and metals or ligands alone at the range of concentrations 15–500 μM. The analysis of apoptotic and necrotic cell death was carried out in three independent experiments according to the manufacturer instructions using Annexin V-FITC apoptosis detection Kit I (BD Pharmingen, USA) and Becton Dickinson LSR II flow cytometer.

The BEAS-2B and A549 were selected for MTT cell metabolic activity test according to manufacturer instructions using EZcount MTT Cell Assay kit (HiMedia, India). The exponentially growing A549 and BEAS-2B cells (1 × 10^5^/ml) were incubated with above metal complexes and their components alone at 7–250 μM for 48 hours. All samples were tested in three independent experiments using Microplate Reader TECAN Infinite 200 PRO (Tecan Group Ltd., Switzerland).

### Analysis of metal complexes interaction with DNA

The effect of metal complexes on DNA was measured by polymerase chain reaction (PCR) with high resolution melting (HRM) using LightCycler 480 II (Hoffman-LaRoche, Switzerland)^20^. The PCR-HRM was performed according to manufacturer instructions using LightCycler 480 SYBR Green I Master (Hoffman-LaRoche, Switzerland). Sequences of primers used in the reaction: forward 5′-GCT GTT TTC AAA GTG ATT TTG GGA GAA-3′ and reverse 5′-CAC TCA TTT GTT TCA AAT TGG AAT G-3′ for the gene *tlr4* with a amplicon length of 147 bp. After amplification, tested metal complexes at 125 μM or positive control (cisplatin at 5–25 μM) were added to the prepared amplicon and incubated for 1 h at 37 °C and subsequently followed by a melting curve HRM protocol. HRM ramps were generated by acquiring fluorescence data at the temperature ramp of 37 °C to 95 °C at 0.1 °C intervals. HRM curve analysis was performed by using the LightCycler 480 Gene Scanning Software (version 1.5).

### Analysis of metal complexes interactions with proteins

The changes in secondary structure of endolysin and lysozyme due to the interaction with complexes were tested using circular dichroism method. Circular dichroism (CD) was measured in the far-UV region (the range between 260 and 195 nm) using a J-815CD spectrometer (Jasco, Japan). The experiments were done in 10 mM sodium phosphate buffer (pH 7.4). The concentration of the endolysin was 1 μM at 20 °C. Quartz 1 cm path length cells (Helma) were used for all CD experiments. The recording scan speed was 50 nm/min. 3 scans were run for each sample, and concentration-dependent with increased metal complexes concentrations up to 1:20 molar ratio (protein:complex respectively) were taken. The CD spectra were corrected by subtracting the spectra (as baseline) obtained for complexes without protein. The mean residue ellipticity *θ*^35^ expressed as mdeg × cm^2^ × dmol^−1^, was calculated^36^.

### Analysis of metal complexes interactions with enzymes

**Figure 8:**
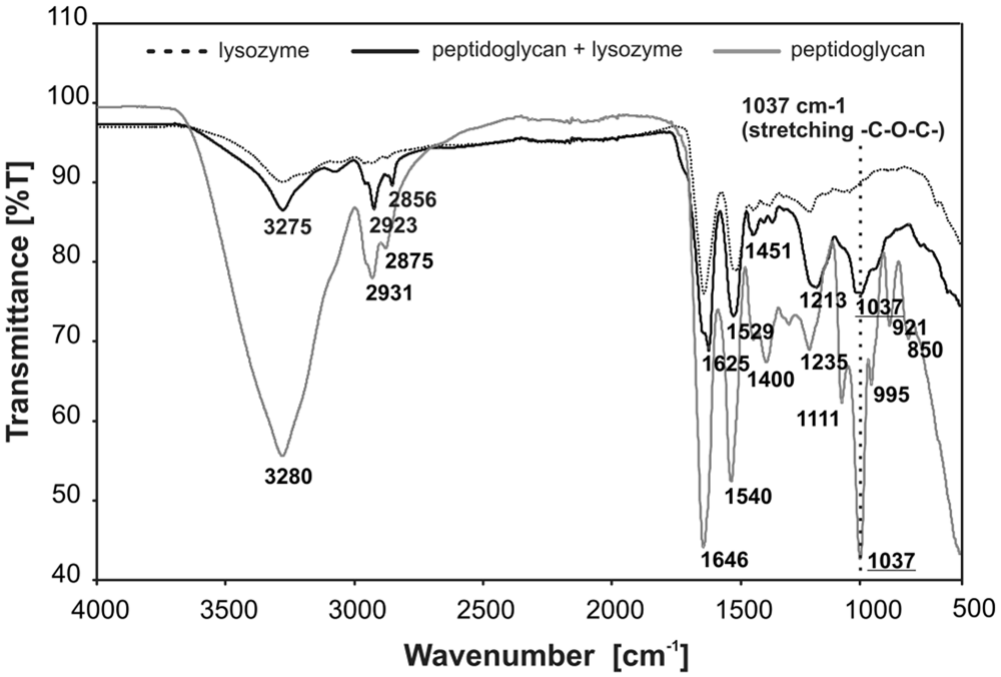
FT-IR spectra (4000–500 cm^−1^) of peptidoglycan treated with lysozyme for 1 h at 37 °C.

**Figure 9:**
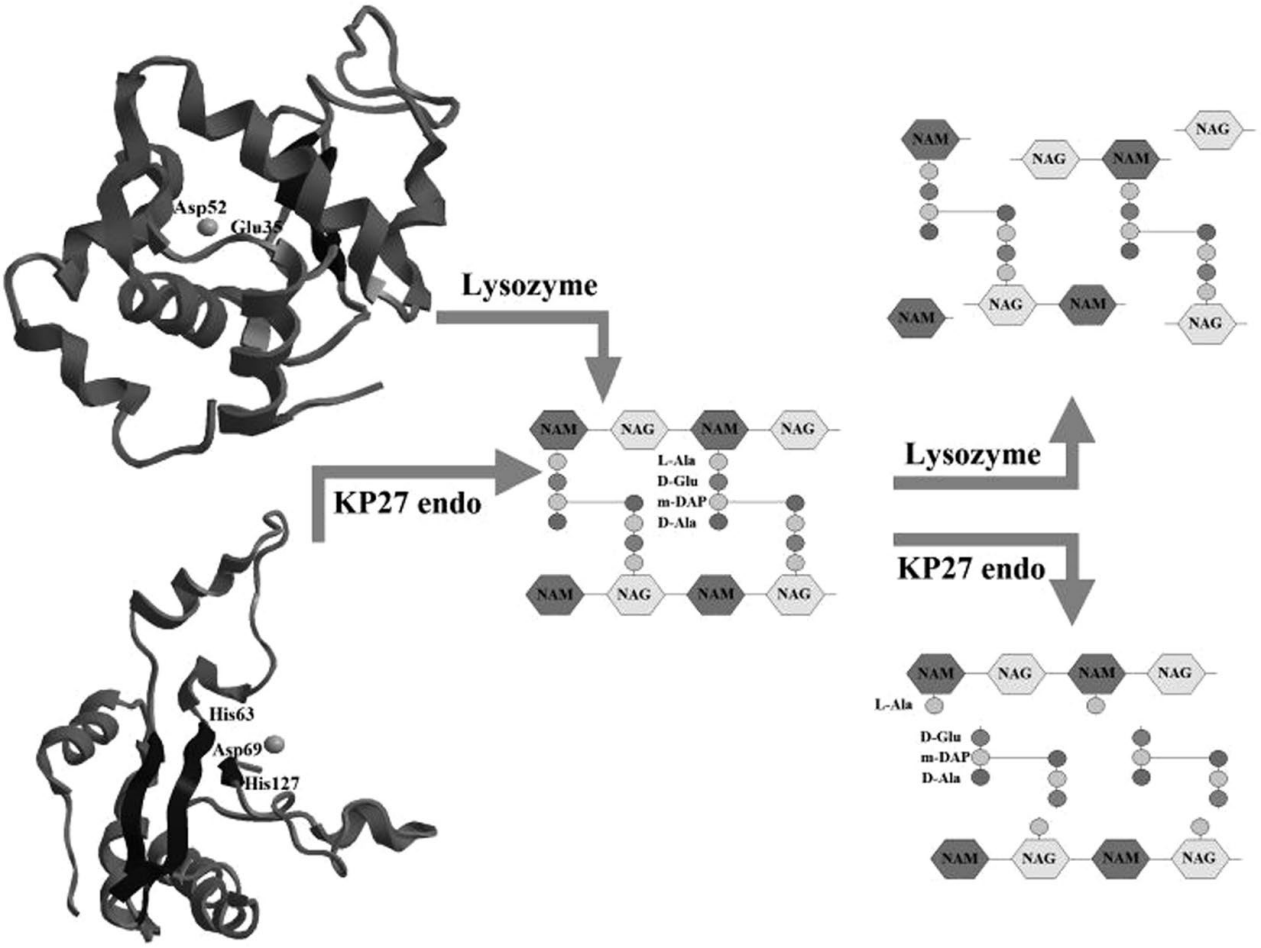
Structures of lysozyme and KP27 endolysin and their specific degradation of peptidoglycan.

PG was isolated from *E. coli* ATCC 8739 cells. Briefly, *E. coli* was cultivated under aerobic conditions in a fermenter (BioStat A, Sartorius Stedim Biotech) in nutrient broth (BTL, Poland) under controlled conditions (37 °C, pH 7.2–7.4, pO_2_ 70–86%). Cells were harvested at the end of the logarithmic growth phase, centrifuged (5000 g, 30 min), washed with distilled water, and lyophilized. PG was isolated in accordance with the method described by Bera *et al*.^35^. It seems to be important to determine the purity of PG suspension in water for lysozyme or endolysin activity testing in the presence of metal complexes. The purity of the PG was tested by FT-IR method using lysozyme as a standard protein with peptidoglycan enzymatic degradation properties. Figure 8 shows FT-IR spectra of PG treated with lysozyme for 1 h at 37 °C. The reduction of -C-O-C- stretching vibrations (1037 cm^−1^) is correlated with PG degradation by lysozyme (hydrolysis of 1,4-beta-linkages between N-acetylmuramic acid and N-acetyl-D-glucosamine residues) (Fig. 9). The results suggest a proper purity of PG for enzymatic assays.

The analysis of metal complexes interactions was performed on peptidoglycan-degrading enzymes (lysozyme and endolysin) as a model (Fig. 9). The degradation of peptidoglycan (PG) by recombinant phage endolysin (KP27 endopeptidase) or lysozyme was measured spectrophotometrically in the presence of Co(II) and Cu(II) complexes with imidazole derivatives. Lysozyme or KP27 endopeptidase were preincubated with metal complexes at 1:5 molar ratio (protein: complex respectively) for 15 min at 37 °C. Secondly, 0.25 mg/ml of PG was added and the kinetics of its degradation was measured at 560 nm for 110 min with 2 min time intervals at 37 °C using Microplate Reader TECAN Infinite 200 PRO (Tecan Group Ltd., Switzerland).

### Antimicrobial properties of metal complexes

The ~2 × 10^5^ cfu/ml of bacterial or fungal culture were grown for 24 h at 37 °C with 5% CO_2_ in stationary culture in Tryptic Soy Broth (Oxoid, USA) or Sabouraud Broth (Oxoid, USA), respectively, in the presence of metal complexes at non-cytotoxic concentrations: 60 µM for [Co(iaa)_2_(H_2_O)_2_]·H_2_O, [Co(1-allim)_6_](NO_3_)_2_ and 30 µM for [Cu(iaa)_2_(H_2_O)], [Cu(1-allim)_4_(NO_3_)_2_]. The Co(II) and Cu(II) ions alone and free ligands (1-allylimidazole and imidazole-4-acetate anion) at 60 µM have been used as controls. Additionally, the microorganisms viability was expressed as colony-forming units (CFU/ml) on Tryptic Soy Agar (Oxoid, USA) or Sabouraud Agar (Oxoid, USA). All samples were measured in three independent experiments.

### Data analysis

The data were analyzed using the Statistica version 10 (StatSoft, Tulsa, OK, USA) software package. P values less than 0.05 were considered statistically significant. The differences were compared by the ANOVA test.
